# Computational inference of a genomic pluripotency signature in human and mouse stem cells

**DOI:** 10.1186/s13062-016-0148-z

**Published:** 2016-09-17

**Authors:** Esra Kurum, Bérénice A. Benayoun, Ankit Malhotra, Joshy George, Duygu Ucar

**Affiliations:** 1 Department of Statistics, University of California, Riverside, Riverside, CA USA; 2 Department of Genetics, Stanford University, Stanford, CA USA; 3 The Jackson Laboratory for Genomic Medicine, 10 Discovery Drive, Farmington, CT 06032 USA

**Keywords:** Pluripotency, Epigenomics, Computational biology, Least absolute shrinkage and selection operator, Embryonic stem cells

## Abstract

Recent analyses of next-generation sequencing datasets have shown that cell-specific regulatory elements in stem cells are marked with distinguishable patterns of transcription factor (TF) binding and epigenetic marks. For example, we recently demonstrated that promoters of cell-specific genes are covered with expanded trimethylation of histone H3 at lysine 4 (H3K4me3) marks (i.e., broad H3K4me3 domains). Moreover, binding of specific TFs, such as OCT4, NANOG, and SOX2, have been shown to play a critical role in maintaining the pluripotency of stem cells. Despite these observations, a systematic exploration of genomic and epigenomic features of stem-cell-specific gene promoters has not been conducted. Advanced machine-learning models can capture distinguishable genomic and epigenomic characteristics of stem-cell-specific promoters by taking advantage of the wealth of publicly available datasets. Here, we propose a three-step framework to discover novel data characteristics of high-throughput next generation sequencing datasets that distinguish pluripotency genes in human and mouse embryonic stem cells (ESCs). Our framework involves: i) feature extraction to identify novel features of genomic datasets; ii) feature selection using a logistic regression model combined with the Least Absolute Shrinkage and Selection Operator (LASSO) method to find the most critical datasets and features; and iii) cross validation with features selected using LASSO method to assess the predictive power of selected data features in distinguishing pluripotency genes. We show that specific epigenetic marks, and specific features of these marks, are enriched at pluripotency gene promoters. Moreover, we also assess both the individual and combined effect of TF binding, epigenetic mark deposition, gene expression datasets for marking pluripotency genes. Our findings are consistent with the existence of a conserved, complex and integrative genomic signature in ESCs that can be exploited to flag important candidate pluripotency genes. They also validate our computational framework for fostering a deeper understanding of genomic datasets in stem cells, in the future, could be extended to study cell-type-specific genomic landscapes in other cell types.

**Reviewers:** This article was reviewed by Zoltan Gaspari and Piotr Zielenkiewicz.

**Electronic supplementary material:**

The online version of this article (doi:10.1186/s13062-016-0148-z) contains supplementary material, which is available to authorized users.

## Findings

In this study, we built a machine-learning framework to identify genomic and epigenomic characteristics of pluripotency gene promoters in embryonic stem cells (ESCs) from human and mouse. Our framework *de novo* identified several predictors previously linked to pluripotency genes: i) an enrichment for known pluripotency regulators (e.g. OCT4 binding), ii) a signature of increased H3K4me3 spread along genomic loci and iii) increased marks of regulation of transcriptional elongation and initiation. These findings are consistent with the existence of a complex and integrative epigenomic signature that, using our model, could be exploited to flag novel important pluripotency genes. Furthermore, the conservation of several features of the pluripotency signature in mouse and human ESCs suggests the existence of common specific constraints for the chromatin environment of genes involved in stem cell pluripotency. We also found that certain characteristics of these datasets are highly correlated, some of which proved very predictive for discriminating stem cell promoters from non-specific promoters, such as the spread (breadth) of H3K4me3 domains found around the gene promoter. Finally, our results revealed the importance of considering additional features of epigenomic signal, like the spread of a histone modification mark over a genomic locus (i.e., peak breadth), or the number of times a gene is marked by a histone mark or bound by a protein. Our computational evaluation of these combinatorial data features showed that, although these features are significantly predictive in marking known pluripotency genes, their predictive power remains modest (AUC~0.7). This implies that pluripotency functions are likely regulated by factors other than the genomic and epigenomic features at gene promoters that we integrated in our models, for instance existence of distal regulatory elements or three-dimensional chromatin interactions between promoter and enhancers. In the future, the predictive power of such models might be expanded with the inclusion of novel types of dataset and further feature engineering. We believe our findings will enable the community to integrate novel and important data characteristics into their studies and, in turn, foster a deeper understanding of specific epigenomic datasets and, perhaps, the hypothesized ‘histone code’ [1].

## Main text

### Introduction

Stem cells have the capability to self-renew, and daughter cells can then differentiate into various tissue lineages. Embryonic stem cells (ESCs) are pluripotent and can give rise to virtually any cell type within the adult organism. In addition to their use as research tools for understanding self-renewal, cellular differentiation and development, ESCs have enormous potential for a range of regenerative cell-based therapies. The pluripotency state of ESCs can be largely mimicked by induced Pluripotent Stem Cells (iPSCs), which are reprogrammed from differentiated cells, and could be a great source of immunogen-free cells for cell based-therapeutics in humans. Elucidating the fundamental mechanisms of pluripotency is critical to assess that differentiated ESC- or iPSC-derived cells are fully committed to their lineage, or that iPSCs have, and maintain, important characteristics of stem cells. Thus, further characterization of the genomic and epigenomic state of genes that play a role in stem cell pluripotency is critical to both understanding the molecular basis of lineage commitment and putting stem cells into safe medical use.

With the decreasing cost of sequencing, large volumes of data are generated via Chromatin Immunoprecipitation followed by high-throughput sequencing (ChIP-seq) technology to profile TF binding and histone modification landscapes in human and mouse embryonic stem cells (ESCs). Studies of genome-wide sequencing datasets have revealed genomic features specific to genes critical for pluripotency. For example, promoters of pluripotency genes are more likely to be marked by broader H3K4me3 domains (i.e., expanded DNA regions marked with H3K4me3) in human and mouse ESCs [2]. Similarly, specific TFs, such as OCT4, NANOG and SOX2, are observed to play critical roles in maintaining the pluripotency functions of stem cells by binding to the promoters of critical genes to regulate their expression [3]. However, most evidence for genomic and epigenomic signatures associated with pluripotency genes to date comes from anecdotal observations [4]. Integrative computational models will enable us to springboard to systematic discoveries by taking advantage of vast amounts of genomic and epigenomic information generated in stem cells and collected in public data repositories.

In this study, we built a statistical framework based on least absolute shrinkage and selection operator (LASSO) and logistic regression [5] to: i) extract novel features from genomic and epigenomic datasets, such as the length of genomic regions with modified histones, which recently was shown to be associated with cell identity [2, 6–8], ii) identify the genomic and epigenomic signatures specific to the promoters of pluripotency genes to discover the genomic signature for pluripotency, and iii) assess the power of this genomic signature in marking known pluripotency genes and discovering novel ones. Through mining of next generation sequencing data in mouse and human ESCs, our computational platform allowed us to quantify the relationship of diverse epigenomic datasets to stem-cell-specific gene promoters, as well as to systematically assess the predictive power of these signatures.

LASSO is a regression method that enables effective feature selection and model interpretation. LASSO-type regularizations have been applied to a wide range of applications in genetics and epigenetics [9–12]. In recent years, it has been successfully applied on learning diverse genomic and epigenomic patterns, including predicting targets of microRNAs from gene expression data [11], gene expression levels from histone modification marks [10], and from copy number variations [9]. In this manuscript, we employed LASSO models to address the following questions in human and mouse ESCs: is there a specific (epi-)genomic signature that marks pluripotency gene promoters in human and mouse stem cells? Can this signature be used to predict novel pluripotency genes? Are features of this pluripotency signature conserved between human and mouse embryonic stem cells? Indeed, we found a genomic signature, predictive of pluripotency in human and mouse embryonic stem cells, which is partially conserved across both organisms. Our main contribution is, in addition to utilizing LASSO-type regularization to effectively address the above-mentioned questions; with our computational models we quantified the predictive power of this signature for identifying important genes for ‘stem cell state’.

### Materials and methods

#### Epigenomic data collection and processing

We collected and analyzed publicly available ChIP-seq datasets assaying histone modification and protein-binding profiles in the human (h) ESC line H1 and in mouse (m) ESCs using public databases. Briefly, we processed the raw ChIP-seq files and filtered out low quality reads with less than 80 % of read length at Phred score >15. Reads were mapped to their respective reference genomes (hg19 for human and mm9 for mouse) using the Bowtie 1 algorithm (version 0.12.7) [13]. ChIP-seq peaks were called with MACS algorithm (version 2.08) [14] (using the “—broad” option for histone marks), and using the corresponding control input data. We assigned peaks to the gene with the closest transcription start site (TSS) using the HOMER toolkit [15]. In hESCs, we processed 25 histone modification marks, including acetylations and methylations, in addition to 43 protein-binding profiles. In mESCs, we analyzed eight epigenetic marks and 59 protein-binding profiles. Additional file 1: Table S1 lists the epigenetic marks and protein-binding datasets in hESCs and mESCs that were analyzed and used in our computational framework, as well as the accession numbers for the raw data used in this analysis.

#### Identification of ‘canonical’ pluripotency genes

In order to learn the pluripotency signature, we compiled a set of “gold-standard” pluripotency genes to compare against genes with no known pluripotency functions. For this purpose, we used data from the ESCAPE database [16], which houses genes with potential pluripotency functions derived from multiple RNAi screening studies. In addition to the ESCAPE dataset, we also incorporated the curated list of known pluripotency genes [17]. In total, we used 397 and 728 putative or known pluripotency genes in hESC and mESC models, respectively. As negative controls, we randomly selected genes that have no known role in pluripotency. This process, in which we used the known pluripotency genes and selected negative controls randomly, was repeated 500 times and, at each run, non-pluripotency genes were selected in equal numbers relative to their pluripotency gene counterparts.

#### Computational framework

**Fig. 1:**
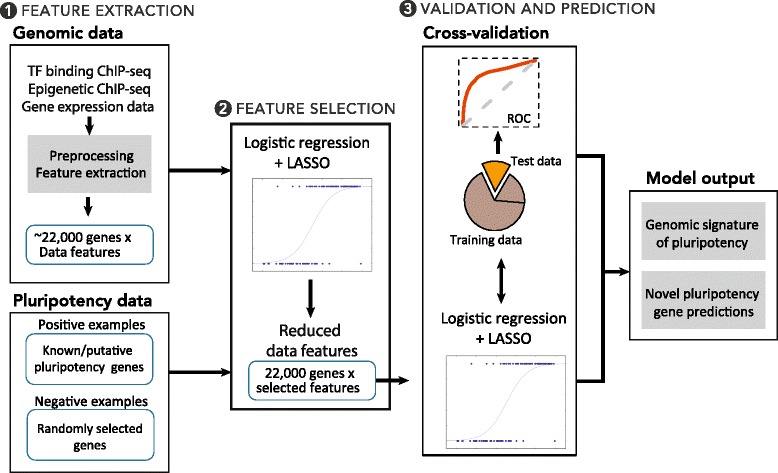
Scheme for our computational framework. A three-step framework to detect genomic and epigenomic characteristics of pluripotency gene promoters in human and mouse embryonic stem cells

We designed a computational platform enabling systematic identification of precise genomic and epigenomic signatures at known pluripotency genes, as well as a means of assessing their power in predicting novel genes that play a critical role in pluripotency. This platform also integrates diverse and complementary datasets in human and mouse stem cells residing in different public databases. Our computational framework is composed of three main steps (Fig. 1):

1. Extracting relevant data features from histone modification and transcription binding ChIP-seq datasets;
2. Selecting the most predictive datasets and their data features (i.e., pluripotency signature) via a multi-step machine-learning model that integrates a logistic regression model with the Least Absolute Shrinkage and Selection Operator (LASSO) method;
3. Assessing the predictive power of the inferred pluripotency signature using cross validation.

This framework enables us to: i) discover genomic signatures specific to pluripotency genes, (i.e., pluripotency signature); ii) infer the contribution of each dataset and data feature(s) to the pluripotency signature; iii) identify similarities and differences between human and mouse pluripotency signatures; and iv) discover novel pluripotency genes through computational prediction.

##### Step 1. Feature extraction

The first step of our framework involves extracting relevant data features from ChIP-seq datasets for human and mouse genes. Using the annotations of peaks to genes for every gene and for each ChIP-seq dataset, we extracted the following features from the data: peak count, distance to closest annotated Transcriptional Start Site (TSS), peak breadth. These features should be able to capture different aspects of the signal with potential biological relevance. First, the number of peaks (i.e., count) in a dataset annotating to a specific gene (e.g., number of OCT4 peaks associated with a gene; defaults to 0 if there are no peaks) will capture the potential existence of multiple discrete bound/marked sites regulating this specific genes, which could have implications on the associated transcriptional response [18]. Secondly, we included the signed distance between the TSS of the annotated gene and the genomic location of the closest peak annotating to it (e.g., distance of the closest OCT4 peak to the gene; in the case of multiple peaks, the closest peak is taken into account) as distance implied to be relevant for genomic signals [3], where negative values mean upstream of the TSS and positive values mean downstream of the TSS. Another important feature is the breadth, which is the genomic spread of the broadest peak marking the gene (e.g., the breadth of H3K4me3 peak marking a gene), a chromatin feature that has been increasingly linked to important biological functions [2, 6–8, 19]. For each ChIP-seq dataset, we extracted the aforementioned features for all genes in hESCs and mESCs, resulting respectively in 76×3 and 63×3 data features for characterizing the genomic and epigenomic landscape. In addition, we also took advantage of gene expression information using RNA-seq datasets from human (GSE30567) and mouse (ERP002100) ESCs in our models. Following the common practice in building LASSO-type models, we used a Z-score normalization approach on all input features including histone modification, protein binding, and gene expression features in order to take care of scaling differences in the data.

##### Step 2. Feature selection

The second step aims to identify data features from epigenetic, TF binding, and gene expression datasets that are specific to pluripotency promoters. A logistic regression model was fit to normalized data features extracted in *Step 1* and was followed by the application of a shrinkage method, the least absolute shrinkage and selection operator (LASSO) [5], which minimizes the sum of squared errors and puts a boundary on the sum of the absolute value of regression coefficients. LASSO was used to identify the best set of features among all histone modification and TF binding data features to predict a pluripotency gene. LASSO typically produces a model that is both easy to interpret and has a conceivably lower prediction error than the full model. The LASSO regression model is also called the L1 regularization model [5]. This means that, unlike the traditional regression models, this model imposes an L1 norm constraint on the regression coefficients. With this constraint, the LASSO model forces the sum of the absolute value of the regression coefficients to be less than a fixed value and shrinks the magnitude of certain coefficients to be set to zero; therefore, chooses a simpler model that does not include those coefficients. By including only important variables in the model, LASSO reduces the number of variables included in the model. Prior to the introduction of LASSO, stepwise selection was widely used to choose important variables; however, this method can only work under certain conditions such as when only a few variables have strong relationships with the outcome.

The LASSO-step in our framework revealed a pluripotency signature from our input TF binding and histone modification datasets. We applied our feature selection method on all: i) epigenetic data features; ii) TF binding data features; and iii) on a combined set of epigenetic and TF binding data features. This enabled us to learn a TF binding signature, an epigenetic signature, and a combined epigenetic and protein binding signature of pluripotency in human and mouse ESCs. We assessed the performance of each LASSO procedure by calculating the true-positive and false-positive rates. To do so, we computed the true-positive and false-positive rates and generated a receiver-operating curve (ROC). A ROC represents the true-positive rate against the false-positive rate at a given threshold setting. A random classifier, with random guesses for a binary classification task, will lead to a diagonal ROC (a.k.a., no-discrimination line) from the bottom-left to the top-right corners. LASSO procedure is fit using glmnet function in ‘glmnet’ package in R statistical software with these two arguments.

##### Step 3. Classification model training

Our platform aims to assess the predictive power of our computationally inferred pluripotency signatures by a 10-fold cross validation procedure that uses the selected features. These features are selected based on the LASSO procedure. For hESCs and mESCs, we built three main types of models: one for each pluripotency signature type (i.e., protein-binding only, histone marks only and combined) using the features extracted in *Step 2*. For each analysis, as discussed above, we built 500 models by randomly selecting a set of negative gene examples for each model building, and predicted the genes with pluripotency signature using each model. At this step, we assessed the number of times we predicted each gene correctly.

### Results

#### Epigenetic and protein-binding profiles constitute a discriminative signature at pluripotency genes

**Fig. 2:**
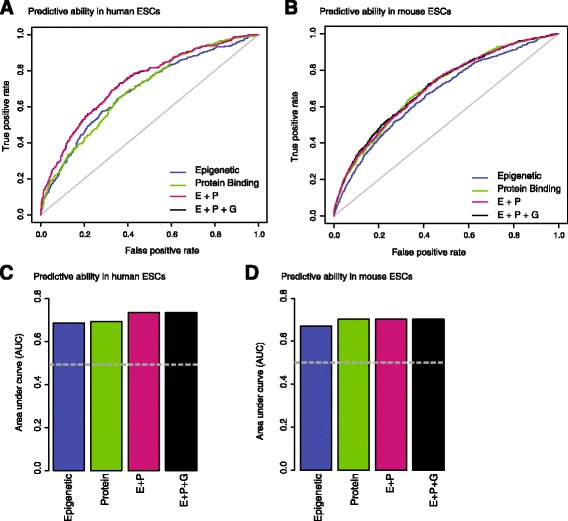
Receiver operating curves (ROCs) and area under curve values (AUCs) to quantify predictive power of our models. ROCs and AUCs show that our computational models can predict pluripotency genes in human (**a**-**c**) and mouse (**b**-**d**) stem-cells better than random expectations

Our models in hESCs and mESCs had significant predictive power as quantified by the Receiver Operator Curves (ROC) and the area under the curve (AUC), which shows the overall classification accuracy of these models (Fig. 2). A random classifier would be expected to have 50 % AUC, which is equivalent to a completely random guess for a binary classification. In contrast, our LASSO models – trained by histone marks only (‘epigenetic’), protein-binding patterns (‘protein binding’), and combined sets of these features (‘E+P’) – could distinguish successfully between pluripotency and non-pluripotency genes >70 % of the time in either hESCs or mESCs. For comparison purposes, we also assessed the added predictive power of gene expression levels by including them into our logistic regression models at *Step 3* (Fig. 2). Our analyses showed that the best scoring models are obtained when histone modifications and protein-binding datasets are integrated, which emphasizes the added value of building integrative models (AUC in hESCs: 0.703; AUC in mESCs: 0.735; Fig. 2c-d). Interestingly, in both cases, incorporating mRNA expression data along with protein binding and epigenetic datasets did not further improve the models’ performances. This observation points out that in the existence of comprehensive epigenetic and protein binding datasets, information in gene expression datasets are redundant.

#### Conserved features of a pluripotency gene signature in human and mouse ESCs

**Fig. 3:**
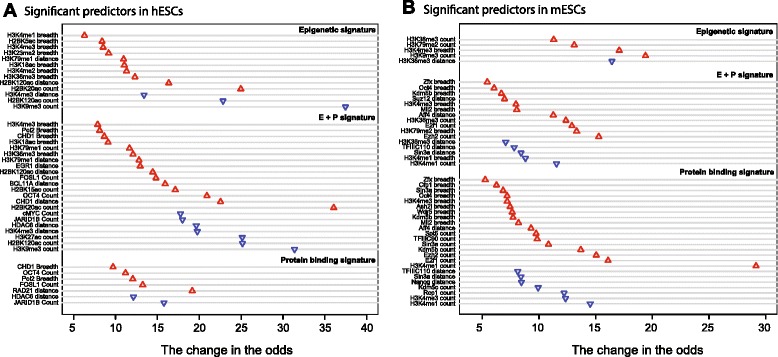
Genomic signatures obtained via LASSO feature selection. Signatures discovered at pluripotency genes in human (**a**) and mouse (**b**) ESCs. Data features enriched at pluripotency gene promoters are depicted with red up-facing triangles; features depleted at pluripotency gene promoters are depicted with blue down-facing triangles

Next, we focused on data features selected using our LASSO procedure to uncover human and mouse pluripotency signatures. The features selected by our LASSO procedure are shown in Fig. 3. Note that gene expression values were not among selected features. Thus, we performed the remainder of our analyses using protein-binding characteristics, histone modifications, and combined models (epigenetic + protein binding). Though the exact features did not always overlap, our analyses indicate that the signature of pluripotency genes in hESCs and mESCs share a conserved enrichment for the binding of specific protein complexes or histone modification regulation.

First, consistent with its role as a master transcriptional regulator of pluripotency [20], we identified OCT4 binding patterns to be important predictors of pluripotency genes. Notably, both breadth and count of this TF were predictive of pluripotency, implying binding of multiple OCT4 proteins over a continuous locus around pluripotency gene promoters.

Extreme H3K4me3 breadth is associated with cell identity genes, which include pluripotency genes in mouse and human ESCs [2, 21]. Consistent with this finding, we found that H3K4me3 breadth is a significant predictor of pluripotency genes both in mESCs (top predictor in the histone modification model) and hESC (top 3rd predictor in the histone modification model). Interestingly, the distance to the closest H3K4me3 domain was a negative predictor of pluripotency in hESCs, implying the importance of having an H3K4me3 domain closer to the TSS. Further supporting the link between H3K4me3 breadth and cell identity, the breadth of deposition of several proteins that are known to associate with H3K4me3 marked nucleosomes, was also predictive for pluripotency both in mESCs and hESCs. These include the JARID1B/Kdm5b H3K4me3 demethylase [22], H3K4me3 trimethyltransferase complex subunits Ash2l, WDR5 and Mll2 [23], and the nucleosome remodeler CHD1, which can bind to H3K4me3 [24]. Independent of their link to H3K4me3, many of these predictive proteins have been shown to modulate ESC pluripotency. Indeed, Chd1 has also been shown to act as an important regulator of pluripotency in mESCs [25]. The presence of Jarid1b/Kdm5b is essential for the ability of mESCs to differentiate properly along the neural lineage [26]. Finally, several members of the H3K4me3 deposition complex have also been directly involved in the maintenance or acquisition of stem cell pluripotency [27–32], which is consistent with their predictive value for pluripotency genes. These published findings support the validity of our approach for identifying a *bona fide* signature of pluripotency genes in mammalian ESCs.

Other features common to mouse and human pluripotency gene–associated signatures include transcriptional elongation or transcriptional activity characteristics, including the H3K36me3 histone mark [33], elongation factor Spt5, binding of RNA Polymerase II (Pol2) or transcription initiation protein TFIIIC. This is in line with findings showing that the H3K36 methyl-transferase Setd2 is required for the differentiation of mESCs along the endoderm lineage [34]. Interestingly, breadth or fold-enrichment of H3K79 methylation, which is another mark of transcriptional elongation, is also predictive according to our models (Fig. 3b). This finding is in agreement with recent findings that identified Dot1l, the enzyme that regulates the deposition of this mark, as an important regulator of mESC pluripotency and reprogramming [35, 36]. Our computational models identified a genomic signature, which suggests that ESCs require a very specific and precise transcriptional regulation mode for maintenance of pluripotency.

In the context of chromatin state, the pluripotency gene signatures we identified were generally enriched for active chromatin states. Our results also revealed that the epigenetic component of the pluripotency signature is generally composed of increased activating relative to repressive epigenetic marks. For example in hESCs, we identified importance of several acetylation marks including acetylation of H2BK5 and H3K18 residues. Similarly, in mESCs, we found that activating marks, including H3K79me2, H3K4me3 and H3K36me3 feature prominently in the epigenetic pluripotency signature. As part of the hESC pluripotency signature, we identified a depletion of H3K9me3 peaks at putative pluripotency promoters, though not captured in mESCs. H3K9me3 is a repressive mark that is thought to target constitutive heterochromatin [37]. Together with the enrichment for active chromatin marks, the reduced number of H3K9me3 in the vicinity of pluripotency genes is consistent with the fact that, as expected, these promoters have active chromatin characteristics in ESCs.

In contrast, our models found increased binding of EZH2 (Enhancer of Zeste homolog 2) at pluripotency gene promoters in mESCs. EZH2 is the catalytic subunit of the PRC2 complex, which is responsible for deposition of the H3K27me3 chromatin mark, a mark traditionally associated with facultative heterochromatin [37]. Though the presence of this repressive complex at pluripotency genes may seem paradoxical, genome-wide analyses have revealed that the PRC2 complex co-localizes with many repressed targets of pluripotency master regulators, OCT4, SOX2, and NANOG, in hESCs [38]. Thus, our models may have captured a subset of developmental genes that must be repressed in ESC to maintain pluripotency, but are poised for subsequent (and requisite) activation during differentiation [39], and may thus represent a key subset of important ‘bivalent’ genes, i.e., genes marked with both activating and repressive epigenetic marks [21].

To understand whether there is a distribution bias for the predictive features, we generated violin plots that represent the distribution of signed distance from the TSS (Additional file 2: Figure S1). These analyses revealed that there are no notable biases in the genomic distribution of these features: the distance is centered around TSS and the location of both TF binding and histone modification marks are evenly distributed in the upstream and downstream of genes. We also observed that most features are close to the TSS (within 10 kb), whereas some can be as far as 100 Kbps. Interestingly, we didn’t observe a significant difference in terms of the location of TF and histone modification features.

#### Different data features contribute to the pluripotency signature

Our feature extraction and selection steps enabled us to study different characteristics of ChIP-seq datasets and assess the importance of each of these characteristics in marking pluripotency gene promoters in stem cells. We found that one of the most robust features of pluripotency associated signatures in our protein binding and histone modification datasets is the number of peaks annotated with pluripotency genes, as well as the spread of the binding over the locus (Fig. 3). For proteins, this implies that the same protein binds at multiple loci around the pluripotency gene promoter. For histone marks, this implies that there are multiple disjoint genomic loci marked with that particular histone mark in the vicinity of the TSS of a pluripotency gene. Most genomic studies reduce the TF binding and epigenetic mark datasets into binary information (i.e., whether there is a TF binding or not). Our results imply that future studies will benefit from taking into consideration not only the existence of a mark or a protein binding event, but also the spread of TF binding or histone modification at a locus.

#### Certain characteristics of epigenetic and protein binding datasets are correlated

To understand the relationships between the different features of ChIP-seq peaks, we calculated the Spearman rank correlation coefficient values between these features using all human and mouse genes with signal in at least one of these datasets. Our analysis showed that the breadth of an epigenetic mark is highly correlated with the number of times that promoter is tagged with that specific histone mark (Additional file 3: Figure S2), which could indicate that i) the experiments were likely not sequenced to saturation or ii). Current peak callers may fail to precisely define boundaries of expanded ChIP-seq peaks and call multiple disjoint peaks in lieu of expanded histone mark deposition. Consistently, we identified the same relationship between the breadth of protein binding peaks and the number of times that a protein is bound to the promoter. To better understand how breadth and count features of histone modification marks are related and how this could impact our models, we calculated the correlation coefficient for the selected datasets (Additional file 4: Table S2). This analysis revealed that count and breadth of histone modification marks are typically positively yet moderately correlated (correlation coefficient ~0.22 for both human and mouse. Moreover, this analysis also revealed that the selected features in our models are typically not highly correlated, consistent with the ability of LASSO to enable the selection of features linked to pluripotency, yet uncorrelated to each other.

#### Candidate pluripotency genes can be predicted computationally

**Fig. 4:**
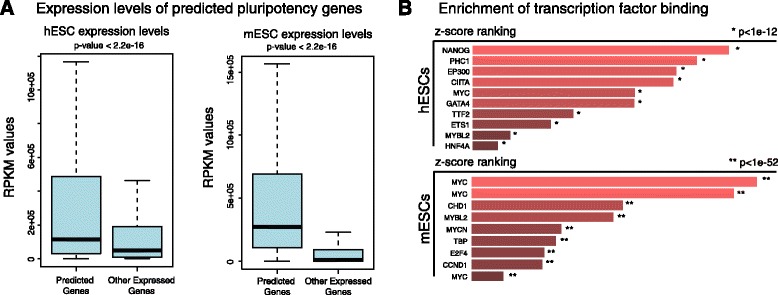
Analyses of predicted embryonic stem cell regulators. **a** Computationally predicted pluripotency genes exhibit elevated expression in human and mouse expression datasets. **b** Motifs enriched in the promoters of predicted pluripotency genes in human and mouse including TFs active in ESCs such as LEF1 and SP1, shades of red represent -log *p*-values of enrichment

Using our predictive models, we next searched for genes with no known role in pluripotency that exhibit our computational signature. In this way, we were able to predict 593 putative pluripotency genes in human and 3211 in mouse ESCs by identifying genes that are robustly predicted as pluripotent in ≥90 % of our resampled models (listed in Additional file 5: Table S3). We identified that 181 of these genes are common between mice and human (Additional file 5: Table S3), which is greater than expected by chance (Fisher overlap *p*-value <2.2e-16). This suggests that the pluripotency network is likely to be conserved between mouse and human ESC. To evaluate whether these predicted genes have elevated gene expression levels in embryonic stem-cell datasets, we first evaluated their mRNA expression levels in ESCs, using publically available RNA-seq datasets. Our analyses showed that these genes are expressed at significantly higher levels in both human and mouse ESCs compared to all other expressed genes (Fig. 4a). However, it is unlikely that our models merely capture the most highly expressed genes in ESCs, because gene expression datasets alone are not as predictive as integrated models obtained from multiple histone mark and protein binding datasets in hESCs (Additional file 6: Figure S3).

Next, we used the EnrichR tool (http://amp.pharm.mssm.edu/Enrichr/) to explore the TF regulatory patterns among the promoters for these genes. We found these genes are indeed enriched in binding of pluripotency related proteins in diverse datasets obtained from public ChIP-seq and ChIP-chip experiments, such as SP1, NANOG and MYC (Fig. 4b), which was consistent with Gene Set Enrichment Analyses (Additional file 7: Figure S4). These observations suggest that our framework can identify genes with potentially important roles in pluripotency functions in ESCs. The list of genes predicted by our models is available as a resource for the stem cell research community in Additional file 5: Table S3.

### Discussion

We built a three-step computational framework to uncover consistent predictors of pluripotency genes in human and mouse ESCs and assess their predictive power. These predictors consist of: i) an enrichment for pluripotency regulators, ii) a signature of increased H3K4me3 spread and iii) increased marks of regulation of transcriptional elongation and initiation. Together, these findings are consistent with the existence of a complex and integrative epigenomic signature that, using our model, can be exploited to flag important pluripotency genes.

We were able to identify common patterns predictive of pluripotency genes in mouse and human ESCs, suggesting conserved pathways may regulate the chromatin landscape of pluripotency genes across species. However, specific features were found to be predictive in only mouse (e.g. Sin3a distance) or human (e.g. H3K9me3 count). The existence of non-overlaping features predictive of pluripotency between mouse and human could result from the fact that some features were only available in one of the species, and that there are some partial cryptic redundancy over these features. However, it may also likely be the result of fundamental differences in the biology of hESC and mESC [40, 41], with in particular hESC being in a less ‘naïve’ state than their murine counterparts. Thus, the differences we observed in the chromatin signature of pluripotency genes may also be a symptom of species differences of or of these divergent states of ‘naïve-ness’, and could help shed light on the mechanisms underlying these differences.

Although we were able to identify features predictive in marking known pluripotency genes, the predictive power of our models remains limited (AUC~0.7), suggesting the existence of other factors for the importance of a gene’s role in pluripotency in addition to the genomic signature at its promoters, for example existence of distal regulatory elements or binding of a long non-coding RNA. In the future, the predictive power of such models might be expanded with the inclusion of novel datasets. Taken together, we believe our findings will enable the community to further integrate novel and important data characteristics into their studies and, in turn, foster a deeper understanding of specific epigenomic datasets and, perhaps, the hypothesized ‘histone code’ [1].

We believe our study can particularly be informative in advancing our understanding of induced pluripotent cells and cancer stem cells. The ‘stem-like’ characteristics of Cancer Stem Cells (CSCs), i.e., self-renewal and proliferation, make cancer treatment even more difficult [42]. Indeed, these cells can remain quiescent for long periods of time and then get reactivated leading to cancer relapse. Therefore, it will be essential to understand the epigenomic characteristics of other types of stem cells such as CSCs and to compare these characteristics to the ones we identified in embryonic stem cells (ESCs). We foresee that our work can be useful in the future in characterizing CSCs in two main directions. First, this genomic signature that we captured (i.e., 181 genes that are predicted to be important for pluripotency in both in human and mouse cells) could be used to assess the pluripotency characteristics of cancer cells. Moreover, with the increase of genomics information in CSCs, similar statistical models of cancer pluripotency can eventually be built and compared to the embryonic stem/pluripotency signature. These efforts might help in identifying CSCs in cancer cells and ultimately be essential in developing therapeutic strategies against chemo-resistant cancers. In addition, our models can be significant in improving our understanding of induced pluripotent stem cells (iPSCs) and in which ways they are similar and different than ESCs. In recent years, a growing compendium of genomic and epigenomic profiling data has started to be generated in human and mouse iPSCs [43]. Together with conserved pluripotency candidates inferred from our models (i.e., 181 genes) as biomarkers, these datasets could be mined to evaluate the degree of achieved pluripotency during the reprogramming process. Moreover, our models could be extended to study the genomic and epigenomic landscape associated with cell-type-specific genes in differentiated cell types. Moreover, recent evidence shows that in addition to cell-type-specific promoters, cell-type-specific enhancers also have distinct epigenomic profiles [19, 44]. Although it is challenging to identify a true set of cell-specific enhancers due to distal promoter-enhancer targeting, with the increasing amount of three-dimensional chromatin-interaction datasets, such as ChIA-PET datasets, this approach could be extrapolated to study the signature at cell-type-specific enhancers in a systematic manner.

## Reviewers’ comments

### Reviewer’s report 1: Zoltan Gaspari

Reviewer summary:

The presented research deals with the identification of pluripotency genes from TF binding, epigenetic signature and gene expression data using a machine-learning approach. I think that the study is both timely and well-conducted and the results are novel and important and the list of putative pluripotency genes merits further investigations. The relatively modest performance of the predictions reveal the importance of further research in the area.

*Authors’ response:* We thank the reviewer for his supportive comments of our study and for highlighting the general interest of our biological findings. We have made a concerted effort to address all of his concerns. We list each criticism and briefly describe how we have addressed it in our revised manuscript. Changes made in the revised manuscript are marked in yellow.

Reviewer recommendations to authors:

Major recommendations:

It might be worth providing a list of the identified pluripotency genes and their relationship in mouse and human (as supplementary material).

*Authors’ response:* We have previously provided a list of genes in human and mouse along with their correct prediction scores (Additional file 5: Table S3). To uncover the relation between human and mouse predictions, we further studied the genes that are frequently predicted as pluripotent in our models (≥90 %). Our analyses revealed that there is statistically significant overlap between these predictions, where 181 of these genes are shared between human and mouse (enrichment *p*-value using fisher’s exact test based on 30 K total genes is <2.2e-16). *We have included this information into our manuscript (page 17) and also provided this list in the updated Additional file*5*: Table S3.*

Is Z-score-based normalization justified in all cases? I mean, there might be bimodal or more complex distance distributions. Please comment on that whether there are such cases and whether these can affect the conclusions.

*Authors’ response:* The purpose of the standardization was to follow the common practice in LASSO-type regularizations. Before employing LASSO regularization, it is recommended to standardize variables so that the solution does not depend on the measurement unit. In addition, the logistic regression model we used in our manuscript does not place any normality assumptions on the covariates. *We clarified this choice in the revised manuscript (page 10).*

Minor recommendations:

According to the description, only the distance from the TSS is considered. Please comment on whether the direction could be important (and whether it would be technically feasible to consider it).

*Authors’ response:* We thank the reviewer for this comment and for giving us an opportunity to clarify the way we processed this data in our models. In our models, we used the signed distance from the TSS, where negative values mean upstream of the TSS and positive values mean downstream of the TSS. *We have clarified this in the manuscript (page 9).*

It might be worth to provide some notes about the precision of the location of the epigenetic marks/TF binding sites with highest found predictive values.

*Authors’ response:* We thank the reviewer for this interesting suggestion. To answer this question, we generated violin plots that represent the distribution of signed distance from the TSS for selected predictive features. *We have included this as Additional file*2*: Figure S1 and discussed these results (page 15) in our revised manuscript*. Our analyses revealed that there aren’t any notable biases in the genomic distribution of these features: the distance is centered around TSS and the location of both TF binding and histone modification marks are evenly distributed in the upstream and downstream of genes. We also observed that most features are close to the TSS within 10 kb, whereas some can be as far as 100 Kbps. Interestingly, we didn’t observe a significant difference in terms of the location of TF and histone modification features.

It could be worth to add a short discussion about the differences between hESCs and mESCs, to what extent might these come from our differential knowledge/data availability and existing biological differences between the organisms.

*Authors’ response:* We thank the reviewer for this suggestion, which will greatly enhance the biological interpretation of our findings. *We have now included a paragraph in the discussion* to address the potential causes of the differences in predictive features between mESCs and hESCs, which we also copy below:

“We were able to identify common patterns predictive of pluripotency genes in mouse and human ESCs, suggesting conserved pathways may regulate the chromatin landscape of pluripotency genes across species. However, specific features were found to be predictive in only mouse (e.g. Sin3a distance) or human (e.g. H3K9me3 count). The existence of non-overlaping features predictive of pluripotency between mouse and human could result from the fact that some features were only available in one of the species, and that there are some partial cryptic redundancy over these features. However, it may also likely be the result of fundamental differences in the biology of hESC and mESC [1, 2], with in particular hESC being in a less ‘naïve’ state than their murine counterparts. Thus, the differences we observed in the chromatin signature of pluripotency genes may also be a symptom of species differences of or of these divergent states of ‘naïve-ness’, and could help shed light on the mechanisms underlying these differences.”

Please provide more details about the relationship between the count and breadth of certain marks. In these cases it could be important to see the exact distribution of the data correlated.

*Authors’ response:* We thank the reviewer for pointing this out. To better understand how breadth and count features of histone modification marks are related and how this could impact our models, we calculated the correlation coefficient for the selected datasets and provided these in *Additional file*4*: Table S2.* This analysis revealed that count and breadth of histone modification marks are typically positively yet moderately correlated (correlation coefficient ~0.22 for both human and mouse. Moreover, this analyses also revealed that the selected features in our models are typically not highly correlated, consistent with the ability of LASSO to enable the selection of features linked to pluripotency, yet uncorrelated to each other (*page 16*).

Please clarify that in Step3 (Classification and model training) you have selected the negative gene set _in addition_ to the list of known pluripotency genes and that this 500 times repeated procedure is the same as mentioned in “Identification of ‘canonical’ pluripotency genes”

*Authors’ response:* Based on the reviewer’s suggestion, we clarified the relation between Step 3 and the “Identification of ‘canonical’ pluripotency genes” sections to *the end of page 7*. “This process, in which we used the known pluripotency genes and selected negative controls randomly, was repeated 500 times and, at each run, non-pluripotency genes were selected in equal numbers relative to their pluripotency gene counterparts.”

We also clarified the relation between these parts by referring to the “Identification of ‘canonical’ pluripotency genes” section *at the end of Step 3 section on page 11.* “For each analysis, as we discussed in the identification of canonical genes section, we built 500 models by randomly selecting a set of negative gene examples for each model building, and predicted the genes with pluripotency signature using each model.”

In Additional file 4: Table S2, please indicate which genes you regard as putative pluripotency genes.

*Authors’ response:* We chose 90 % to be the cutoff value, that is, a gene with a prediction rate that is greater than 90 % is regarded as putative pluripotency gene. *In the revised Additional file*5*: Table S3, we highlighted these genes and included this cutoff criterion at the top of the file. We also included genes that are common between human and mouse into the Additional file*5*: Table S3.*

I would suggest combining all data into a single multi-tab Excel file for easier handling.

*Authors’ response:* Predictions from human and mouse are provided *in the tabs of Additional file*5*: Table S3*. We keep human and mouse predictions in separate tabs, yet provide the common genes between human and mouse in this.

### Reviewer’s report 2: Piotr Zielenkiewicz

Reviewer summary:

I have found the paper very interesting. Authors applied a Lasso method for modelling epigenetic regulation of genes in human and mouse embryonic stem cells (ESCs). It was a good read.

I am, however, confused as the authors did not show what is novel in their approach. The Lasso regression model has already been applied for the construction of microRNA-target regulatory network (see the paper “A Lasso regression model for the construction of microRNA-target regulatory networks”, Bioinformatics. 2011 Sep 1;27(17):2406–13) and for modelling of impact of histone modifications on gene activity (see “Modelling epigenetic regulation of gene expression in 12 human cell types reveals combinatorial patterns of cell-type-specific genes”, IET Syst Biol. 2014 Jun;8(3):104–15). The authors seem to be unaware of these contributions. This leads to a confusion about the relation of the Lasso algorithm they used to the previous applications. On the positive side, it should be mentioned that the Authors’ approach properly predicts gene activity.

This is very strong part of the paper.

*Authors’ response:* We thank the referee for his thoughtful and constructive comments and his appreciation of our work. We have made a concerted effort to address all of his concerns. We list each criticism and briefly describe how we have addressed it.

I believe the Authors should in more detail discuss the biological significance of their study i.e. describe what is the impact of expanded trimethylation of histone H3 at lysine 4 on activity of apoptotic genes and cellular metabolism.

The paper “The Warburg effect version 2.0: Metabolic reprogramming of cancer stem cells, Menendez et al. 2013, Cell Cycle. 2013 Apr 15;12(8):1166–79” can be particularly useful in this respect.

*Authors’ response:* We thank the reviewer for this great suggestion. *We have included a detailed discussion* on how this study can be expanded and utilized to understand epigenomics characteristics of induced pluripotent stem cells and cancer stem cells in the discussion section of our manuscript. We also copied this below:

“We believe our study can particularly be informative in advancing our understanding of induced pluripotent cells and cancer stem cells. The ‘stem-like’ characteristics of Cancer Stem Cells (CSCs), i.e., self-renewal and proliferation, make cancer treatment even more difficult [3]. Indeed, these cells can remain quiescent for long periods of time and then get reactivated leading to cancer relapse. Therefore, it will be essential to understand the epigenomic characteristics of other types of stem cells such as CSCs and to compare these characteristics to the ones we identified in embryonic stem cells (ESCs). We foresee that our work can be useful in the future in characterizing CSCs in two main directions. First, this genomic signature that we captured (i.e., 181 genes that are predicted to be important for pluripotency in both in human and mouse cells) could be used to assess the pluripotency characteristics of cancer cells. Moreover, with the increase of genomics information in CSCs, similar statistical models of cancer pluripotency can eventually be built and compared to the embryonic stem/pluripotency signature. These efforts might help in identifying CSCs in cancer cells and ultimately be essential in developing therapeutic strategies against chemo-resistant cancers.

In addition, our models can be significant in improving our understanding of induced pluripotent stem cells (iPSCs) and in which ways they are similar and different than ESCs. In recent years, a growing compendium of genomic and epigenomic profiling data has started to be generated in human and mouse iPSCs [4]. Together with conserved pluripotency candidates inferred from our models (i.e., 181 genes) as biomarkers, these datasets could be mined to evaluate the degree of achieved pluripotency during the reprogramming process.”

Reviewer recommendations to authors:

References to previous similar contributions should be added. The discussion section should be extended to contain the biological significance of the results.

*Authors’ response:* We thank the reviewer for alerting us to these interesting studies and for pointing out the strengths of our study; we have included references to similar studies and included a brief discussion to highlight the novelty of our work in the introduction.

We agree with the reviewer that LASSO regression model has been used in literature several times. We have included a summary of the literature on LASSO-type regularizations to the introduction of our paper and highlighted in which ways our study is similar and different than some of these previous studies. Similar to previous studies published in the literature, we also employed the traditional LASSO regression model, yet we differ by utilizing LASSO-type regularization to address a new set of questions. *We included the following paragraph to the introduction of the revised manuscript to summarize some of the literature on LASSO-type models and to clarify the contribution of our paper*:

“LASSO is a regression method that enables effective feature selection and model interpretation. LASSO-type regularizations have been applied to a wide range of applications in genetics and epigenetics [5–8]. In recent years, it has been successfully applied on learning diverse genomic and epigenomic patterns, including predicting targets of microRNAs from gene expression data [7], gene expression levels from histone modification marks [6], and from copy number variations [5]. In this manuscript, we employed LASSO models to address the following questions in human and mouse ESCs: is there a specific (epi-)genomic signature that marks pluripotency gene promoters in human and mouse stem cells? Can this signature be used to predict novel pluripotency genes? Are features of this pluripotency signature conserved between human and mouse embryonic stem cells? Indeed, we found a genomic signature, predictive of pluripotency in human and mouse embryonic stem cells, which is partially conserved across both organisms. Our main contribution is, in addition to utilizing LASSO-type regularization to effectively address the above-mentioned questions; with our computational models we quantified the predictive power of this signature for identifying important genes for ‘stem cell state’.”

Minor issues:

The Lasso method (the knowledge of which is rare) should be described to at least the same level of detail as ROC curves (known to everybody).

*Authors’ response:* We thank the reviewer for his useful feedback; *we have included the following detailed description of LASSO models to the end of page 10 in our manuscript.*

“The LASSO regression model is also called the L1 regularization model, that is, unlike the traditional regression models; this model imposes an L1 norm constraint on the regression coefficients. With this constraint, the LASSO model forces the sum of the absolute value of the regression coefficients to be less than a fixed value and shrinks the magnitude of certain coefficients to be set to zero; therefore, chooses a simpler model that does not include those coefficients. By including only important variables in the model, LASSO reduces the number of variables included in the model. Prior to the introduction of LASSO, stepwise selection was widely used to choose important variables; however, this method can only work under certain conditions such as when only a few variables have strong relationships with the outcome.”

## Additional files

Additional file 1: Table S1. Accession codes for epigenetic and protein-binding datasets used in our computational framework. (XLSX 62 kb) Additional file 2: Figure S1. Distribution of distance to TSS for selected features (A) human and (B) mouse. (PDF 376 kb) Additional file 3: Figure S2. Epigenetic and protein binding dataset correlations. Correlations between a subset of epigenetic and protein binding data features (assessed by Spearman correlation coefficient) indicate that certain datasets and features are highly correlated with each other in human (A-B-C) and mouse (D-E-F) embryonic stem cells. (PDF 11059 kb) Additional file 4: Table S2. Pairwise correlation coefficients between selected histone modification marks including count and breadth data features. (XLSX 42 kb) Additional file 5: Table S3. Predicted stemness regulators in human and mouse ESCs from the logistic regression models with LASSO (E+P). (XLSX 834 kb) Additional file 6: Figure S3. Receiver operating curves (ROCs) and area under curve values (AUCs) to quantify predictive power of our models including models built with gene expression data alone in human (A-C) and mouse (B-D). (PDF 1675 kb) Additional file 7: Figure S4. Gene Set Enrichment Analyses of genes ranked with respect to the number of times each gene is predicted as ‘pluripotent’ in LASSO models. (PDF 153 kb)

## Abbreviations

AUC: Area under the curve
ESC: Embryonic stem cell
LASSO: Least absolute shrinkage and selection operator
ROC: Receiver operating curve
TF: Transcription factor
TSS: Transcriptional start site
