# Supplementary figures and images for: Computational inference of a genomic pluripotency signature in human and mouse stem cells
Source: Biol Direct. 2016 Sep 17;11:47. doi: 10.1186/s13062-016-0148-z (PMC5027095; doi:10.1186/s13062-016-0148-z)

## **A** Selected hESC datasets

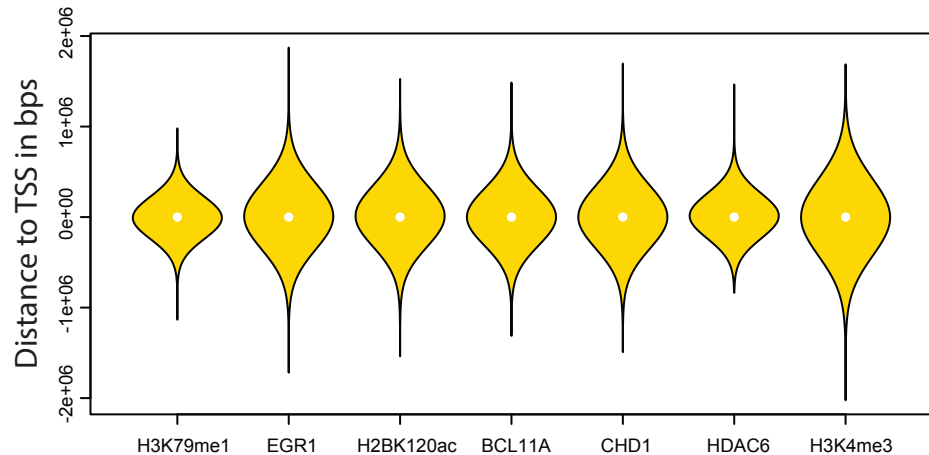

## **B** Selected mESC datasets

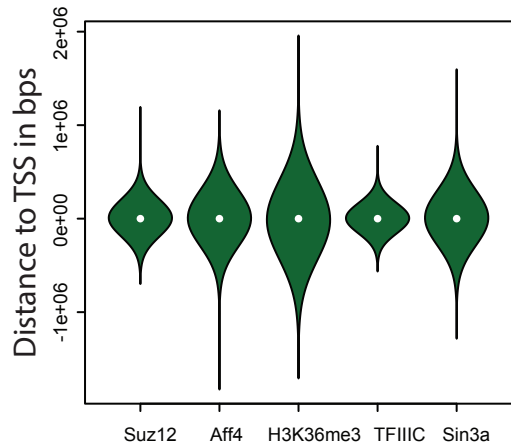

Supplement: Additional file 2: Figure S1. — Distribution of distance to TSS for selected features (A) human and (B) mouse. (PDF 376 kb) [file 13062_2016_148_MOESM2_ESM.pdf]
