# Supplementary material for: Computational inference of a genomic pluripotency signature in human and mouse stem cells
Source: Biol Direct. 2016 Sep 17;11:47. doi: 10.1186/s13062-016-0148-z (PMC5027095; doi:10.1186/s13062-016-0148-z)
Supplement: Additional file 3: Figure S2. — Epigenetic and protein binding dataset correlations. Correlations between a subset of epigenetic and protein binding data features (assessed by Spearman correlation coefficient) indicate that certain datasets and features are highly correlated with each other in human (A-B-C) and mouse (D-E-F) embryonic stem cells. (PDF 11059 kb) [file 13062_2016_148_MOESM3_ESM.pdf]

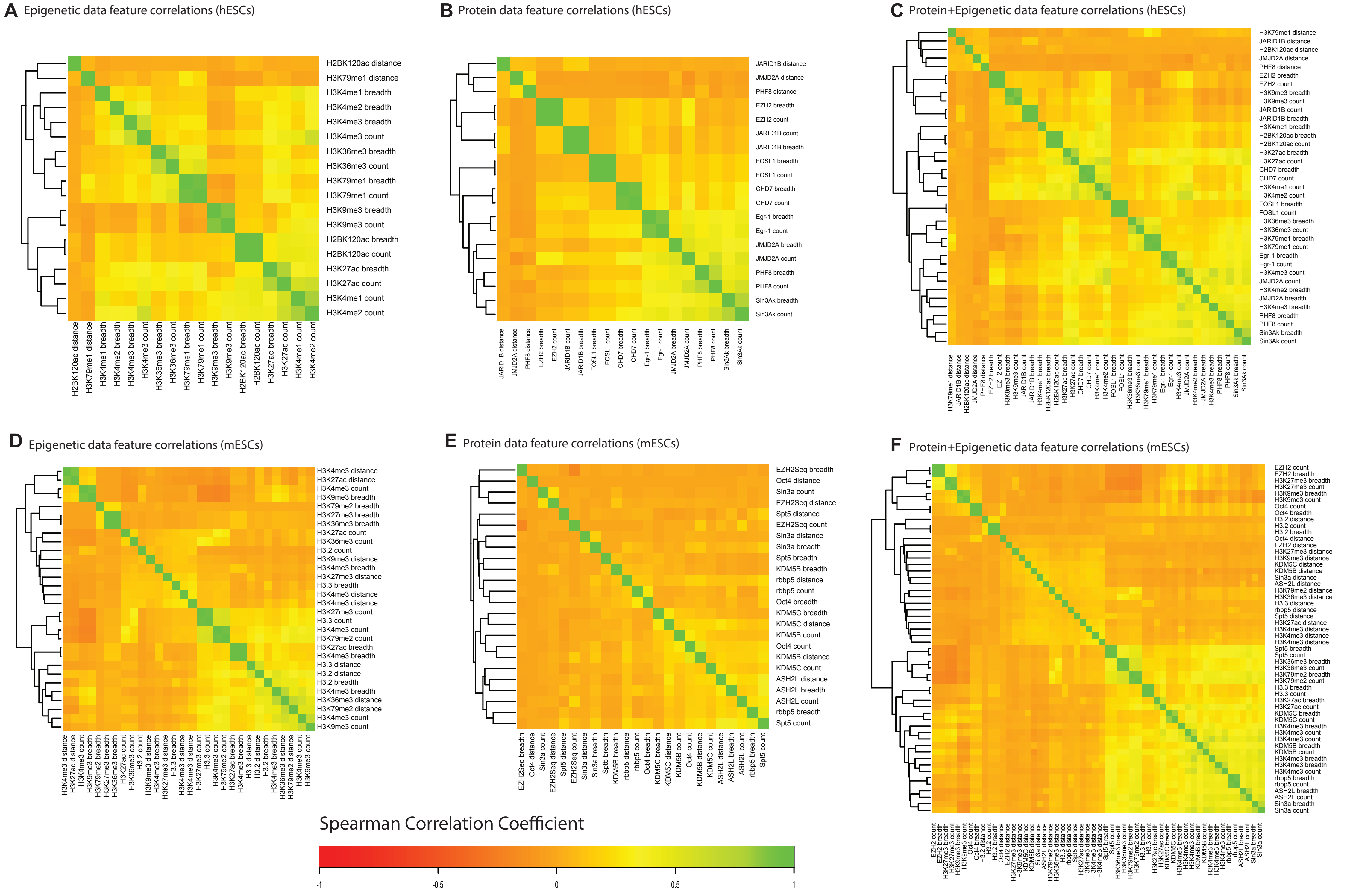
A Epigenetic data feature correlations (hESCs)
H2BK120ac distance
H3K79me1 distance
H3K4me1 breadth
H3K4me2 breadth
H3K4me3 breadth
H3K4me3 count
H3K36me3 breadth
H3K36me3 count
H3K79me1 breadth
H3K79me1 count
H3K9me3 breadth
H3K9me3 count
H2BK120ac breadth
H2BK120ac count
H3K27ac breadth
H3K27ac count
H3K4me1 count
H3K4me2 count
B Protein data feature correlations (hESCs)
JARID1B distance
JMJD2A distance
PHF8 distance
EZH2 breadth
EZH2 count
JARID1B count
JARID1B breadth
FOSL1 breadth
FOSL1 count
CHD7 breadth
CHD7 count
Egr-1 breadth
Egr-1 count
JMJD2A breadth
JMJD2A count
PHF8 breadth
PHF8 count
Sin3Ak breadth
Sin3Ak count
C Protein+Epigenetic data feature correlations (hESCs)
H3K79me1 distance
JARID1B distance
H2BK120ac distance
JMJD2A distance
PHF8 distance
EZH2 breadth
EZH2 count
H3K9me3 breadth
H3K9me3 count
JARID1B count
JARID1B breadth
H3K4me1 breadth
H2BK120ac breadth
H2BK120ac count
H3K27ac breadth
H3K27ac count
CHD7 breadth
CHD7 count
H3K4me1 count
H3K4me2 count
FOSL1 breadth
FOSL1 count
H3K36me3 breadth
H3K36me3 count
H3K79me1 breadth
H3K79me1 count
Egr-1 breadth
Egr-1 count
H3K4me3 count
JMJD2A count
H3K4me2 breadth
JMJD2A breadth
H3K4me3 breadth
PHF8 breadth
PHF8 count
Sin3Ak breadth
Sin3Ak count
D Epigenetic data feature correlations (mESCs)
H3K4me3 distance
H3K27ac distance
H3K4me3 count
H3K9me3 breadth
H3K79me2 breadth
H3K27me3 breadth
H3K36me3 breadth
H3K27ac count
H3K36me3 count
H3.2 count
H3K9me3 distance
H3K4me3 breadth
H3K27me3 distance
H3.3 breadth
H3K4me3 distance
H3K4me3 distance
H3K27me3 count
H3.3 count
H3K4me3 count
H3K79me2 count
H3K27ac breadth
H3K4me3 breadth
H3.3 distance
H3.2 distance
H3.2 breadth
H3K4me3 breadth
H3K36me3 distance
H3K79me2 distance
H3K4me3 count
H3K9me3 count
E Protein data feature correlations (mESCs)
EZH2Seq breadth
Oct4 distance
Sin3a count
EZH2Seq distance
Spt5 distance
EZH2Seq count
Sin3a distance
Sin3a breadth
Spt5 breadth
KDM5B breadth
rbbp5 distance
rbbp5 count
Oct4 breadth
KDM5C breadth
KDM5C distance
KDM5B count
Oct4 count
KDM5B distance
KDM5C count
ASH2L distance
ASH2L breadth
ASH2L count
rbbp5 breadth
Spt5 count
F Protein+Epigenetic data feature correlations (mESCs)
EZH2 count
EZH2 breadth
H3K27me3 breadth
H3K27me3 count
H3K9me3 breadth
H3K9me3 count
Oct4 count
Oct4 breadth
H3.2 distance
H3.2 count
H3.2 breadth
Oct4 distance
EZH2 distance
H3K27me3 distance
H3K9me3 distance
KDM5C distance
KDM5B distance
Sin3a distance
ASH2L distance
H3K79me2 distance
H3K36me3 distance
H3.3 distance
rbbp5 distance
Spt5 distance
H3K27ac distance
H3K4me3 distance
H3K4me3 distance
H3K4me3 distance
Spt5 breadth
Spt5 count
H3K36me3 breadth
H3K36me3 count
H3K79me2 breadth
H3K79me2 count
H3.3 breadth
H3.3 count
H3K27ac breadth
H3K27ac count
KDM5C breadth
KDM5C count
H3K4me3 breadth
H3K4me3 count
H3K4me3 count
KDM5B breadth
KDM5B count
H3K4me3 breadth
H3K4me3 breadth
H3K4me3 count
rbbp5 breadth
rbbp5 count
ASH2L breadth
ASH2L count
Sin3a breadth
Sin3a count
Spearman Correlation Coefficient
-1
-0.5
0
0.5
1
