# Supplementary material for: Computational inference of a genomic pluripotency signature in human and mouse stem cells
Source: Biol Direct. 2016 Sep 17;11:47. doi: 10.1186/s13062-016-0148-z (PMC5027095; doi:10.1186/s13062-016-0148-z)
Supplement: Additional file 6: Figure S3. — Receiver operating curves (ROCs) and area under curve values (AUCs) to quantify predictive power of our models including models built with gene expression data alone in human (A-C) and mouse (B-D). (PDF 1675 kb) [file 13062_2016_148_MOESM6_ESM.pdf]

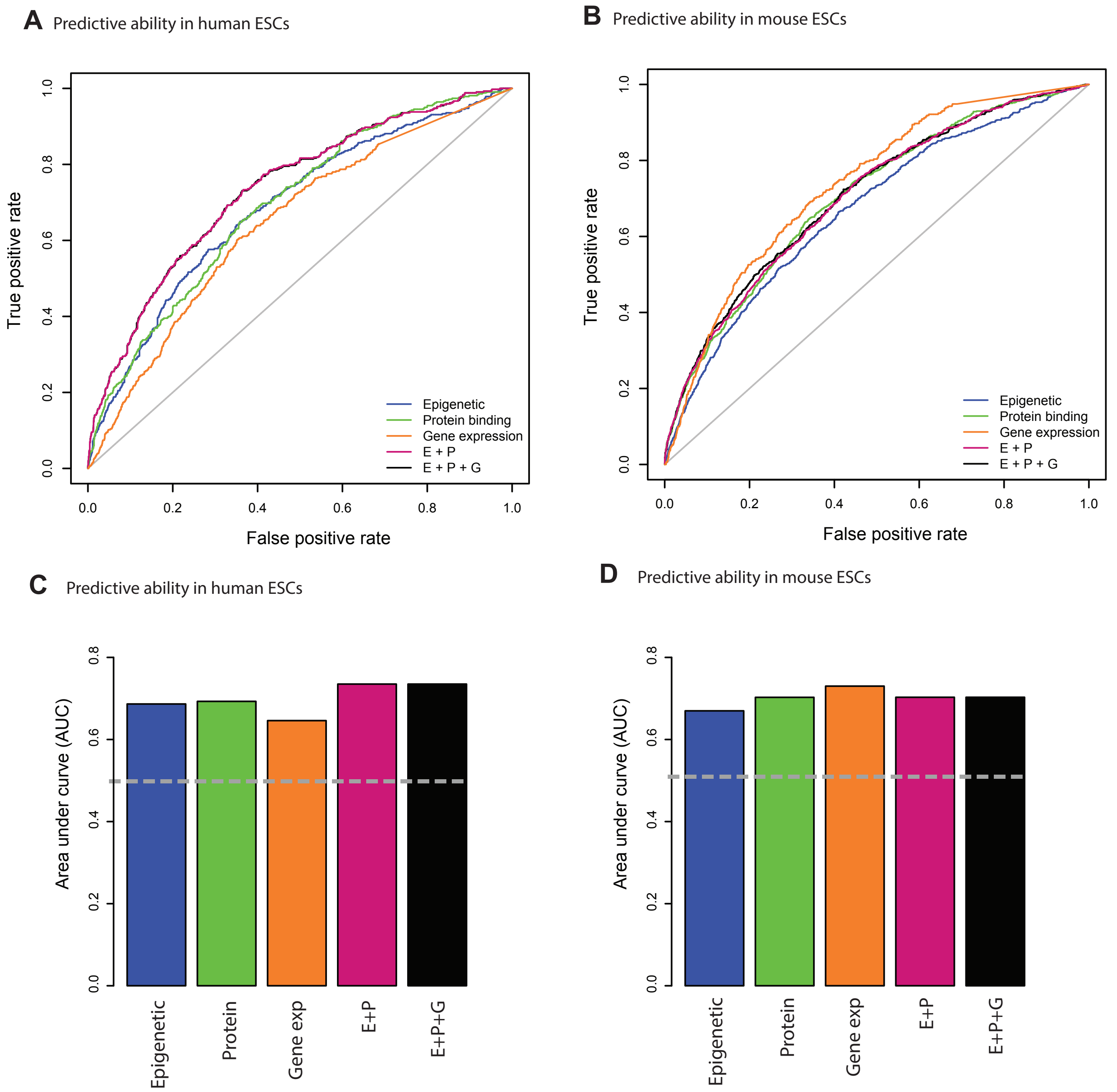

A Predictive ability in human ESCs
True positive rate
False positive rate
0.0
0.2
0.4
0.6
0.8
1.0
Epigenetic
Protein binding
Gene expression
E + P
E + P + G
B Predictive ability in mouse ESCs
True positive rate
False positive rate
Epigenetic
Protein binding
Gene expression
E + P
E + P + G
C Predictive ability in human ESCs
Area under curve (AUC)
Epigenetic
Protein
Gene exp
E+P
E+P+G
D Predictive ability in mouse ESCs
Area under curve (AUC)
Epigenetic
Protein
Gene exp
E+P
E+P+G
