# Supplementary material for: Computational inference of a genomic pluripotency signature in human and mouse stem cells
Source: Biol Direct. 2016 Sep 17;11:47. doi: 10.1186/s13062-016-0148-z (PMC5027095; doi:10.1186/s13062-016-0148-z)

A GSEA for predicted genes in hESCs

Gene Set	FDR q-value	FWER q-value
MYC_TARGETS_V1	0.032	0.032
MTORC1_SIGNALING	0.027	0.052
E2F_TARGETS	0.024	0.072
MITOTIC_SPINDLE	0.033	0.127
MYC_TARGETS_V2	0.027	0.13
WNT_BETA_CATENIN_SIGNALING	0.022	0.132
G2M_CHECKPOINT	0.019	0.132
NOTCH_SIGNALING	0.018	0.135
UNFOLDED_PROTEIN_RESPONSE	0.030	0.236
PROTEIN_SECRETION	0.029	0.258
TGF_BETA_SIGNALING	0.033	0.316
TNFA_SIGNALING_VIA_NFKB	0.042	0.404

B GSEA results for MYC target gene list

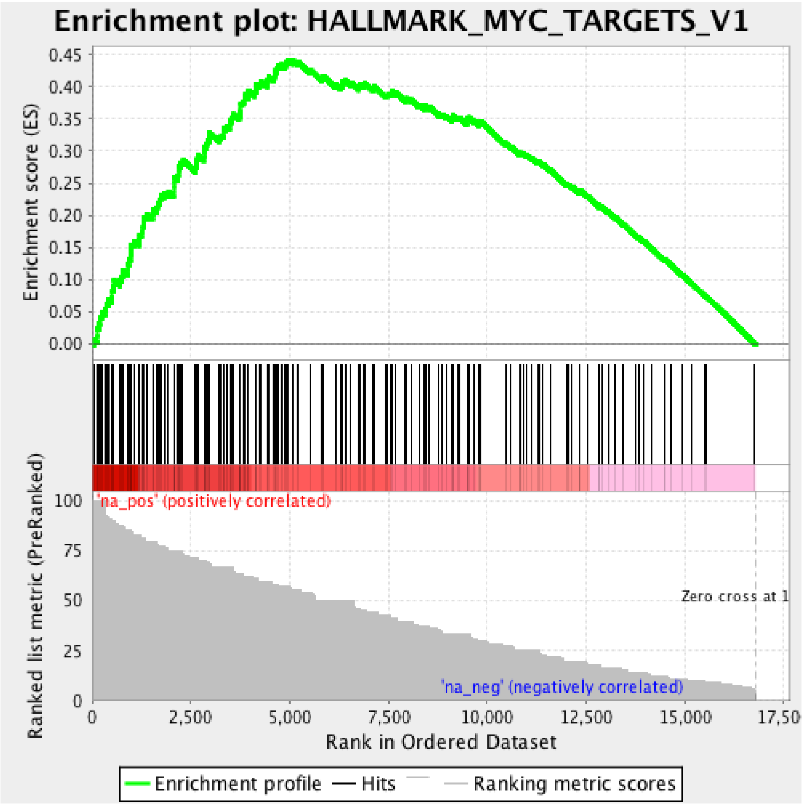

Supplement: Additional file 7: Figure S4. — Gene Set Enrichment Analyses of genes ranked with respect to the number of times each gene is predicted as ‘pluripotent’ in LASSO models. (PDF 153 kb) [file 13062_2016_148_MOESM7_ESM.pdf]
